# Supplementary material for: mTORC2 sustains thermogenesis via Akt‐induced glucose uptake and glycolysis in brown adipose tissue
Source: EMBO Mol Med. 2016 Jan 15;8(3):232–46. doi: 10.15252/emmm.201505610 (PMC4772955; doi:10.15252/emmm.201505610)
Supplement: Supplementary file 1 — Appendix [file EMMM-8-232-s001.pdf]

mTORC2 sustains thermogenesis via Akt-induced glucose uptake and glycolysis in BAT

Verena Albert, Kristoffer Svensson, Mitsugu Shimobayashi, Marco Colombi, Sergio Muñoz, Veronica Jimenez, Christoph Handschin, Fatima Bosch, Michael N. Hall

Appendix

Table of contents

Appendix Table S1
Appendix Table S2

Appendix Table S1. Primer sequences used for qRT-PCR

Target	forward primer (5'-3')	reverse primer (5'-3')
**CACT**	CTGCGCCCATCATTGGA	CAGACCAAACCCAAAGAAGCA
**CPT-1β**	ATCATGTATCGCCGCAAACT	CCATCTGGTAGGAGCACATGG
**Dio2**	GAGGAAGGAAGAAGAGGAAGCAA	TTCTTCCAGTGTTTTGGACA TGC
**D-loop**	GGTTCTTACTTCAGGGCCATCA	GATTAGACCCGTTACCATCGAGAT
**HKII**	AAAACCAAGTGCAGAAGGTTGAC	GAACCGCCTAGAAATCTCCAGAA
**LCAD**	CCAGCTAATGCCTTACTTGGAGA	GCAATTAAGAGCCTTTCCTGTGG
**Ndufv1**	CTTCCCCACTGGCCTCAAG	CCAAAACCCAGTGATCCAGC
**PGC-1α**	GAGAATGAGGCAAACTTGCTAGCG	TGCATGGTTCTGAGTGCTAAGACC
**RFP**	GCGGCCACTACACCTGCGAC	TCGGCGTGCTCGTACTGCTC
**RPL0**	CTGCTGAACATGCTGAACATCTC	CTTCAGGGTTATAAATGCTGCCG
**TBP**	TGCTGTTGGTGATTGTTGGT	CTGGCTTGTGTGGGAAAGAT
**UCP1**	TGATGAAGTCCAGACAGACAGTG	TTATTCGTGGTCTCCCAGCATAG

Appendix Table S2. p-values for statistical tests.

Figure	p-value			
	**control vs. AdRiKO 22°C**	**control vs. AdRiKO 4°C**	**control 22°C vs. 4°C**	**AdRiKO 22°C vs. 4°C**
**2D**	0.00004 (***)	n.d.	n.d.	n.d.
**2F**	n.d.	1h: 0.04 (*)<br>2h: 0.04 (*)<br>4h: 0.006 (**)<br>5h: 0.000003 (***)<br>6h: 0.00004 (***)<br>7h: 0.00002 (***)<br>8h: 0.0000006 (***)	n.d.	n.d.
**3B**	n.s.	0.0001 (***)	0.00000003 (###)	0.0000004 (###)
**3C**	n.s.	n.s.	0.0001 (###)	0.00002 (###)
**3F**	n.s.	0.0096 (**)	0.03 (#)	0.006 (##)
**4A**	PGC-1α: 0.0008 (***)	n.s.	UCP1: 0.00006 (###)<br>Dio2: 0.00007 (###)<br>PGC-1α: 0.0006 (###)	UCP1: 0.04 (#)<br>Dio2: 0.0002 (###)<br>PGC-1α: 0.001 (##)
**4C**	n.s.	n.s.	CACT: 0.04 (#)<br>LCAD: 0.0096 (##)	CACT: 0.02 (#)<br>LCAD: 0.02 (#)
**4G**	n.s.	n.s.	0.003 (##)	0.0008 (###)
**4H**	n.s.	0.01 (*)	0.0000002 (###)	0.000001 (###)
**5A**	n.s.	0.01 (*)	0.00001 (###)	0.02 (#)
**5B**	n.s.	0.01 (*)	0.003 (##)	n.s.
**5F**	n.s.	0.02 (*)	0.004 (##)	n.s.
**5G**	n.s.	n.s.	0.003 (##)	n.s.
	**control vs. AdRiKO AAV-empty**	**control vs. AdRiKO AAV-HKII or AAV-Akt2^{S474D}**	**control AAV-empty vs. AAV-HKII or AAV-Akt2^{S474D}**	**AdRiKO AAV-empty AAV-HKII or AAV-Akt2^{S474D}**
**6A**	n.s.	n.s.	0.00000096 (###)	0.000000004 (###)
**6B**	0.005 (**)	n.s.	0.001 (##)	0.01 (#)
**6C**	0.02 (*)	n.s.	n.s.	0.01 (#)
**6D**	1h: 0.00003 (*a*)<br>2h: 0.046 (*a*)<br>3h: 0.02 (*a*,*)	1h: 0.03 (*b*)	n.s.	2h: 0.02 (*d*)<br>3h: 0.02 (*d*,#)
**6F**	0.001 (**)	0.008 (**)	n.s.	0.01 (#)
**6G**	0h: 0.0085 (*a*)<br>1h: 0.004 (*a*)<br>3h: 0.02 (*a*,*)	1h: 0.01 (*b*)<br>3h: 0.03 (*b*,*)	n.s.	0h: 0.01 (*d*)<br>3h: 0.03 (*d*,#)
**6H**	0.01 (*)	n.s.	n.s.	n.s.
	**control vs. AdRiKO veh.**	**control vs. AdRiKO NE**	**control veh. vs. NE**	**AdRiKO veh. vs. NE**
**EV1E**	n.s.	0.01 (*)	0.04 (#)	n.s.
	**control vs. AdRiKO 22°C**	**control vs. AdRiKO 4°C**	**control 22°C vs. 4°C**	**AdRiKO 22°C vs. 4°C**
**EV1F**	Akt-pS473: 0.002 (**)	Akt-pS473: 0.00006 (***)<br>mTOR-pS2481: 0.005 (**)	Akt-pS473: 0.008 (##)	n.s.
**EV1H**	light phase: 0.01 (*)	n.d.	n.d.	n.d.
**EV1I**	n.d.	7h: 0.03 (*)<br>8h: 0.03 (*)<br>10h: 0.001 (**)	n.d.	n.d.
**EV1J**	n.d.	0.02 (*)	n.d.	n.d.
**EV3A**	n.s.	0.04 (*)	0.002 (##)	0.002 (##)
**EV3B**	0.01 (*)	n.s.	0.008 (##)	0.002 (##)
**EV3C**	n.s.	raptor-pS792: 0.004 (**)	n.s.	raptor-pS792: 0.00009 (###)<br>ACC-pS79: 0.03 (#)

n.d.=not determined, n.s.=not significant (p>0.05)
