# Supplementary material for: mTORC2 sustains thermogenesis via Akt‐induced glucose uptake and glycolysis in brown adipose tissue
Source: EMBO Mol Med. 2016 Jan 15;8(3):232–46. doi: 10.15252/emmm.201505610 (PMC4772955; doi:10.15252/emmm.201505610)
Supplement: Supplementary file 2 — Expanded View Figures PDF [file EMMM-8-232-s002.pdf]

Expanded View Figures

Figure EV1. AdRiKO mice do not display alterations in body weight, plasma IGF-1 and locomotor activity.

A Immunoblot analysis of BAT and sWAT of AdRiKO and control mice housed at 22°C for the indicated proteins.
B Body weight of AdRiKO and control mice housed at 22°C [n = 14 (control), n = 12 (AdRiKO)].
C Body composition of AdRiKO and control mice housed at 22°C (n = 18/group).
D Plasma IGF-1 levels in AdRiKO and control mice housed at 22°C [n = 11 (control), n = 9 (AdRiKO)].
E Quantification of Akt-pS473 band intensity relative to total Akt band intensity shown in Fig 2B (n = 3/group).
F Quantification of Akt-pS473 and mTOR-pS2481 band intensity relative to total Akt or total mTOR band intensity shown in Fig 2C (n = 6/group).
G Immunoblot analysis of sWAT of AdRiKO and control mice housed at 22 or 4°C for 2 h for the indicated proteins (n = 6/group, each lane represents a mix of 3 mice).
H Locomotor activity of AdRiKO and control mice housed at 22°C (n = 13/group).
I Body temperature loss of AdRiKO and control mice upon cold exposure with *ad libitum* access to food [n = 11 (control), n = 10 (AdRiKO)].
J Cold-induced shivering of AdRiKO and control mice housed at 4°C for 4 h (n = 6/group).

Data information: Data represent mean ± SEM. Statistically significant differences between AdRiKO and control mice were determined with unpaired Student's *t*-test and indicated with asterisks (*$P < 0.05$; **$P < 0.01$; ***$P < 0.001$). Statistically significant differences between temperatures or treatments were determined with unpaired Student's *t*-test and are indicated with a number sign ($^{\#}P < 0.05$; $^{\#\#}P < 0.01$). The exact *P*-value for each significant difference can be found in Appendix Table S2.

 © 2016 The Authors

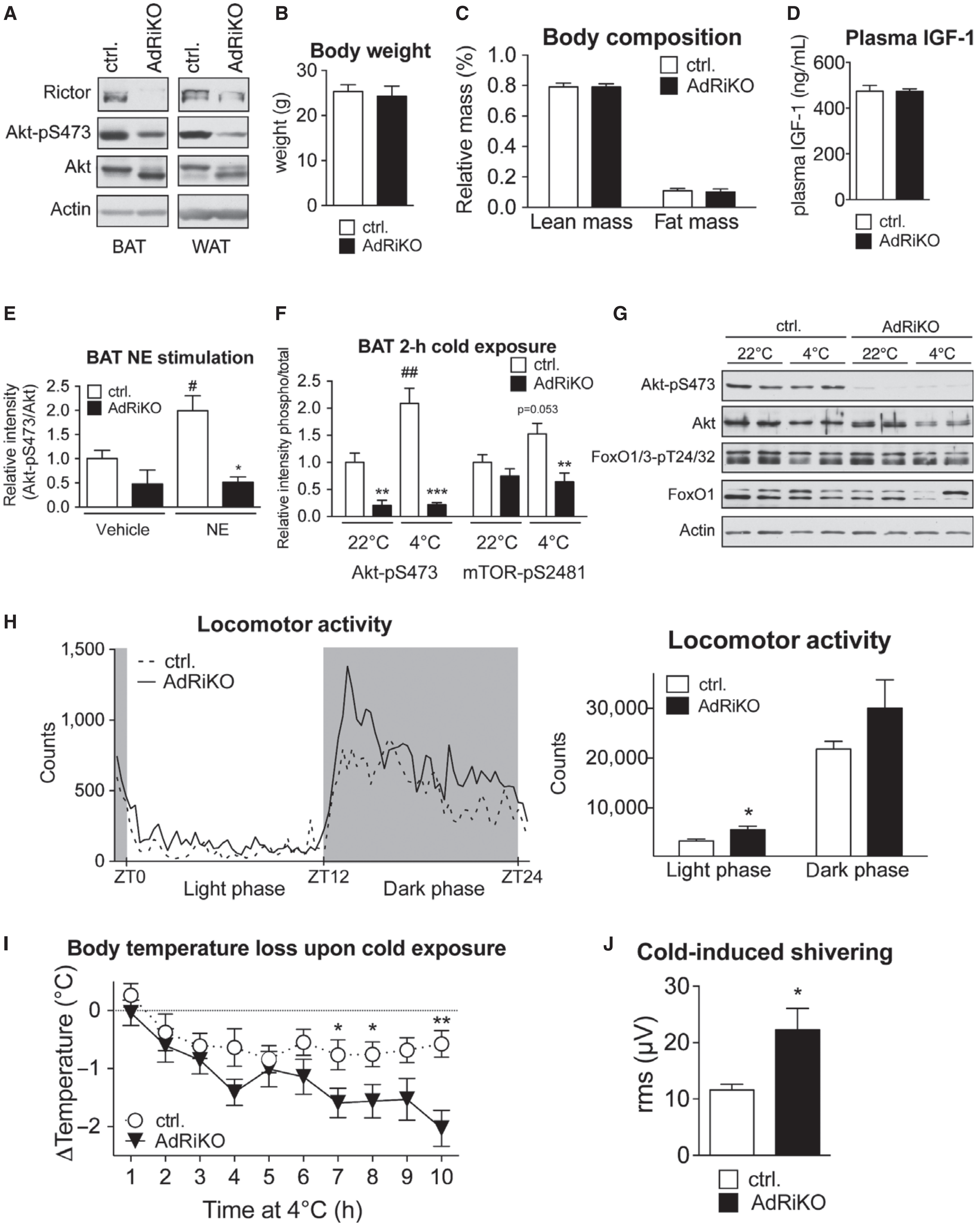
A
ctrl.
AdRiKO
ctrl.
AdRiKO
Rictor
Akt-pS473
Akt
Actin
BAT
WAT
B
Body weight
weight (g)
ctrl.
AdRiKO
C
Body composition
Relative mass (%)
Lean mass
Fat mass
ctrl.
AdRiKO
D
Plasma IGF-1
plasma IGF-1 (ng/mL)
ctrl.
AdRiKO
E
BAT NE stimulation
Relative intensity (Akt-pS473/Akt)
ctrl.
AdRiKO
Vehicle
NE
F
BAT 2-h cold exposure
Relative intensity phospho/total
ctrl.
AdRiKO
p=0.053
22°C
4°C
Akt-pS473
mTOR-pS2481
G
ctrl.
AdRiKO
22°C
4°C
Akt-pS473
Akt
FoxO1/3-pT24/32
FoxO1
Actin
H
Locomotor activity
ctrl.
AdRiKO
Counts
ZT0
Light phase
ZT12
Dark phase
ZT24
Locomotor activity
Counts
Light phase
Dark phase
I
Body temperature loss upon cold exposure
ΔTemperature (°C)
ctrl.
AdRiKO
Time at 4°C (h)
J
Cold-induced shivering
rms (μV)
ctrl.
AdRiKO

Figure EV1.

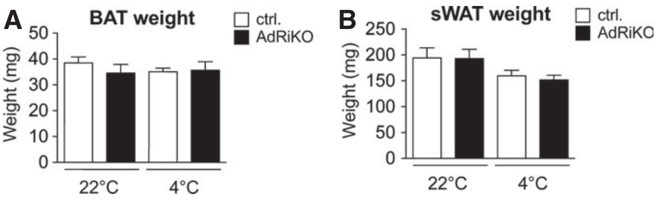

Figure EV2. mTORC2 in adipose tissue does not affect BAT and sWAT weight.

A BAT weight of AdRiKO and control mice housed at 22 or 4°C for 8 h (n = 6/group). Data represent mean ± SEM.

B sWAT weight of AdRiKO and control mice housed at 22 or 4°C for 8 h (n = 6/group). Data represent mean ± SEM.

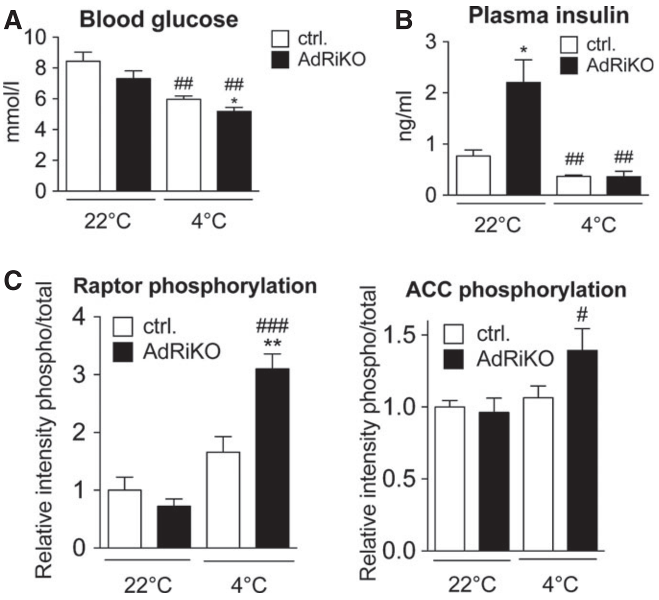

Figure EV3. Cold exposure decreases blood glucose and circulating insulin.

A Blood glucose of AdRiKO and control mice housed at 22 or 4°C for 8 h (n = 7/group).

B Plasma insulin of AdRiKO and control mice housed at 22 or 4°C for 8 h (n = 6/group).

C Quantification of raptor-pS792 and ACC-pS79 band intensity relative to total raptor or total ACC band intensity shown in Fig 5C (n = 6/group).

Data information: Data represent mean ± SEM. Statistically significant differences between AdRiKO and control mice were determined with unpaired Student's t-test and are indicated with asterisks (*$P < 0.05$; **$P < 0.01$). Statistically significant differences between temperatures were determined with unpaired Student's t-test and are indicated with a number sign ($^{\#}P < 0.05$; $^{\#\#}P < 0.01$; $^{\#\#\#}P < 0.001$). The exact P-value for each significant difference can be found in Appendix Table S2.

© 2016 The Authors

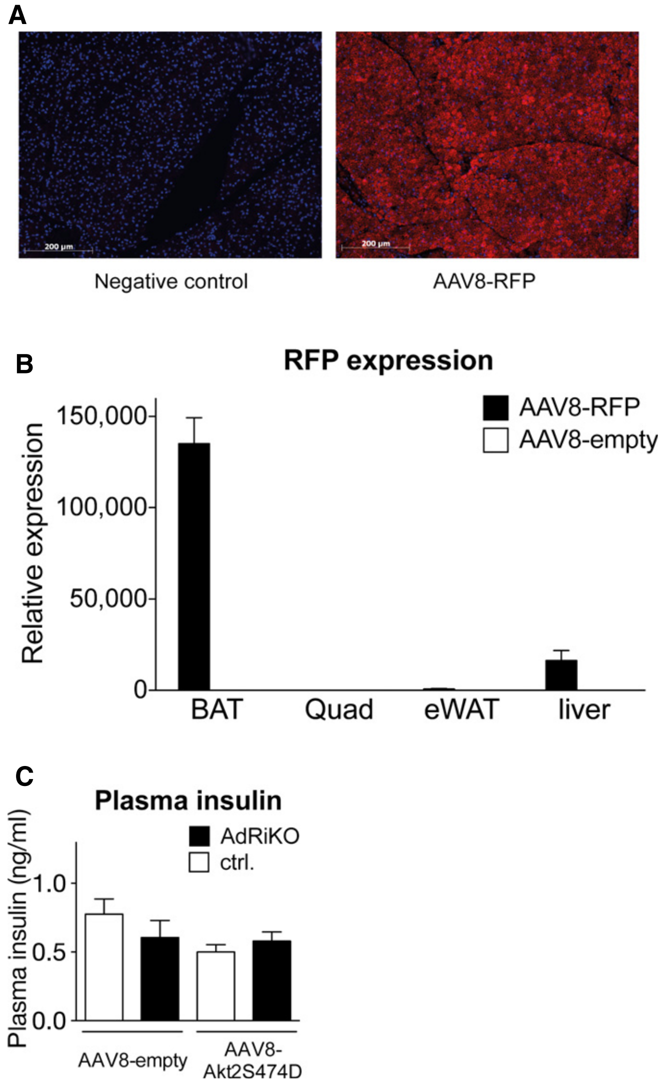

Figure EV4. Intra-BAT injection of AAV targets genes of interest specifically to BAT.

A Representative immunostainings for RFP of BAT from control mice infected with either AAV8-RFP or AAV8-empty (n = 4/group).

B RFP mRNA expression in BAT, liver, quadriceps, and WAT of control mice infected with either AAV8-RFP or AAV8-empty (n = 4/group).

C Plasma insulin of AdRiKO and control mice infected with either AAV8-Akt2^{S474D} or AAV8-empty housed at 4°C for 4 h [n = 7 (control AAV8-null), n = 6 (AdRiKO AAV8-null), n = 6 (control AAV8-Akt^{S474D}), n = 6 (AdRiKO AAV8-Akt^{S474D})].

Data information: Data represent mean ± SEM.

© 2016 The Authors
